# Improving the diagnosis of active tuberculosis: a novel approach using magnetic particle-based chemiluminescence LAM assay

**DOI:** 10.1186/s12890-024-02893-2

**Published:** 2024-02-27

**Authors:** Yan Li, Zhiwei Ru, Hongxia Wei, Ming Wu, Guihua Xie, Jianrong Lou, Xiang Yang, Xilin Zhang

**Affiliations:** 1 Leide Biosciences Co., Ltd, Guangdong, China; 2 Foshan Fourth People’s Hospital, Guangdong, China

**Keywords:** Tuberculosis (TB), Lipoarabinomannan (LAM), Chemiluminescence assay(CLIA)

## Abstract

**Objectives:**

Tuberculosis (TB) is a significant global health concern, given its high rates of morbidity and mortality. The diagnosis using urine lipoarabinomannan (LAM) primarily benefits HIV co-infected TB patients with low CD4 counts. The focus of this study was to develop an ultra-sensitive LAM assay intended for diagnosing tuberculosis across a wider spectrum of TB patients.

**Design & Methods:**

To heighten the sensitivity of the LAM assay, we employed high-affinity rabbit monoclonal antibodies and selected a highly sensitive chemiluminescence LAM assay (CLIA-LAM) for development. The clinical diagnostic criteria for active TB (ATB) were used as a control. A two-step sample collection process was implemented, with the cutoff determined initially through a ROC curve. Subsequently, additional clinical samples were utilized for the validation of the assay.

**Results:**

In the assay validation phase, a total of 87 confirmed active TB patients, 19 latent TB infection (LTBI) patients, and 104 healthy control samples were included. Applying a cutoff of 1.043 (pg/mL), the CLIA-LAM assay demonstrated a sensitivity of 55.2% [95%CI (44.13%~65.85%)], and a specificity of 100% [95%CI (96.52%~100.00%)], validated against clinical diagnostic results using the Mann-Whitney U test. Among 11 hematogenous disseminated TB patients, the positive rate was 81.8%. Importantly, the CLIA-LAM assay consistently yielded negative results in the 19 LTBI patients.

**Conclusion:**

Overall, the combination of high-affinity antibodies and the CLIA method significantly improved the sensitivity and specificity of the LAM assay. It can be used for the diagnosis of active TB, particularly hematogenous disseminated TB.

**Supplementary Information:**

The online version contains supplementary material available at 10.1186/s12890-024-02893-2.

## Introduction

Prior to the emergence of coronavirus disease 2019 (COVID-19), tuberculosis (TB) was the leading cause of death among infectious diseases. According to the World Health Organization’s (WHO) TB report, approximately one-quarter of the world’s population (1.7 billion people) is infected with M. tuberculosis [1]. Developing new diagnostic methods is crucial for early detection, treatment, and preventing the transmission of TB.

Lipoarabinomannan (LAM), a unique component of the lipid-rich mycobacterial cell wall, serves as a highly specific antigen that is excreted in the urine and has the potential to be used as a diagnostic marker for TB testing [2, 3]. The Alere Determine TB LAM antigen assay (AlereLAM) is currently the only product recommended by the WHO for the detection of LAM in urine samples [2], but it only has moderate sensitivity (40–60%) in HIV co-infected patients with low CD4 counts, and low sensitivity (10–20%) in active TB (ATB) only patients [4, 5]. Therefore, there is a need to improve the sensitivity of the LAM assay for HIV-negative TB patients.

The low concentration of LAM in the urine of HIV-negative TB patients is probably the cause of the low sensitivity of the AlereLAM. We hypothesized that utilization of high-affinity antibodies combined with a high-sensitivity assay could improve the sensitivity. In this study, we aimed to describe the novel approach of the assay and calculate the performance of the test.

## Materials and methods

### Reagent and instrument

Magnetic beads were purchased from JSR, Japan (Cat# MS300). EDC was from Pierce, USA (Cat# 77,149). NHS was from Whdsbio, China (Cat# 199293-83-9). MES was from Sigma, USA (Cat#M2933). CLIA instrument was from Keysmile, China (SMART 500 S). LAM antigen was from CNpair, China. BeyoECL plus was from Beyotime, China (#P00185). Protein Ladder, 10 to 250 kDa was purchased from ThermoFisher, USA (Cat# 26,619).

### Crude TB LAM preparation

Cultured M. tb H37Rv cells were autoclaved, washed in PBS, and then broken up by sonication at 180 W for 15 min on ice. The lysate was centrifuged at 20,000 g for 30 min to pellet any unbroken cells (26). The lysis supernatant was named as crude TB LAM.

### Rabbit monoclonal antibody development

Rabbit monoclonal antibodies were developed by LDEBIO (Guangzhou, China). Briefly, rabbits (ages 3–4 months, New Zealand White strain) were immunized with the crude TB LAM antigen for four times. The spleen tissues of the immunized rabbits were used to amplify the rabbit antibody variable domains by PCR. A phage ScFv library was then prepared and screened with commercial LAM antigen, and the ScFv genes of positive phages were sequenced (26). After gene synthesis and cloning, full-length antibodies were expressed in 293 T cells and purified using a Protein-A affinity column. One pair of LAM antibodies was selected for the assay, and their purity and activity were confirmed by SDS-PAGE and Western blot.

### SDS-PAGE and Western blot analysis of the expressed antibodies

The 12% SDS-PAGE gels were poured, run, and stained with Coomassie Blue. About 5ug antibody samples/well were boiled in non-reducing sample buffers (5% SDS, 20% glycerol, 125 mM Tris–HCl, pH 6.8, 0.1% bromophenol blue) and reducing sample buffer (non-reducing sample buffers plus 10mM DTT) for 3 min before loading.

Commercial LAM antigens 0.5, 1, 2 µg, and 10 ul crude TB LAM were loaded into the SDS–PAGE wells (12% polyacrylamide gels) and electro-transferred onto a nitrocellulose membrane. After blocking with 1% milk in PBST, the nitrocellulose membrane reacted with 0.5 ug/ml biotinylated LAM antibody for one hour, then incubated with SA-HRP for 30 min. Then, membrane was incubated with 10 ml BeyoECL plus reagent for 2 min. Drained membrane was wrapped in plastic wrap and exposed to x-ray film. Please refer to Table 1 for abbreviations.

**Table 1:** List of abbreviations

Abbreviation	Full name
TB	Tuberculosis
LAM	Lipoarabinomannan
CLIA	Chemiluminescence assay
COVID-19	Corona Virus Disease 2019
WHO	World Health Organization
USA	The United States of America
PCR	Polymerase chain reaction
ROC	Receiver Operating Characteristic
PAGE	Polyacrylamide gel electrophoresis
HPLC	High Performance Liquid Chromatography
SD	Standard Deviation
MTX	5-methylthio-d-xylofuranose
EPTB	Extrapulmonary disease
ICU	Intensive Care Unit
Conc.	Concentration
NC membrane	Nitrocellulose filter membrane
EDC	1-ethyl-3-[3-dimethylaminopropyl]carbodiimide hydrochloride
NHS	N-Hydroxysuccinimide
KD	kDa
SA	Streptavidin
HRP	Horseradish Peroxidase
AE	Acridinium ester
BLI	Bio-Layer Interferometry
EPTB	Extrapulmonary disease of TB
ECL	Enhanced chemiluminescence
DTT	dithiothreitol
LTBI	Latent Tuberculosis Infection
ATB	Active Tuberculosis

### Antibodies kinetics analysis

Antibodies’ kinetic affinity was measured by GATOR (ProbeLife), which detects the real-time interaction between antibodies and antigens based on the principle of the Bio-Layer Interferometry (BLI) method. The affinity detection process includes equilibration, capture, binding, dissociation, and regeneration. Briefly, antibody molecules were first captured onto the probe, then antigen LAM was diluted into a serial concentration and reacted with the antibody on the probe. The Gator detector detected changes in the thickness and density of the film formed by LAM binding or dissociation and measured the interaction between the antibodies and the LAM molecular.

### Sample collection

This study is dedicated to assay development and employs a two-phase sample collection approach. In the initial phase, our goal is to gather a strategically minimal yet statistically significant number of active tuberculosis (ATB) patient samples (50), along with an equal number of healthy samples, to establish a cutoff value for the LAM assay. The second phase focuses on validating the sensitivity and specificity of the LAM assay, utilizing approximately 100 ATB cases and an equivalent number of healthy samples for a comparative T-test analysis.

The inclusion criteria for ATB patients align with the diagnostic criteria outlined in “Diagnosis for Pulmonary Tuberculosis (WS 288–2017)“ [1] and “Classification of Tuberculosis (WS196-2017)“ [2]. Healthy participants, who underwent routine physical examinations with no history of tuberculosis exposure or symptoms, constitute the comparison group. LTBI patients are identified through a positive interferon-gamma release assay (IGRA) result, negative chest radiographs, and negative clinically diagnosed methods. Hematogenous disseminated tuberculosis is clinically confirmed based on the classical miliary pattern observed on chest radiography or CT scans.

Exclusions encompass patients undergoing tuberculosis treatment, as well as those with unqualified urine samples, such as heavy proteinuria or impurities. Adhering to the principles of the Declaration of Helsinki, the study received approval from the Ethics Committee of the Fourth People’s Hospital of Foshan (No. 2,022,008). Healthy participants from the physical examination department, suspected tuberculosis patients, and in-hospital tuberculosis patients with a negative HIV status visiting the Fourth People’s Hospital of Foshan were recruited for the study.

For sample handling, morning urine samples underwent centrifugation at 3000 rpm for 10 min to remove debris. The resulting supernatants were transferred to new tubes and stored at -20 °C for transportation and centralized testing.

### Chemiluminescent LAM assay development and cutoff value determination

Capture antibody crosslinked to magnetic bead: The carboxyl group of 5 mg magnetic beads was activated by 5 mg/ml EDC and 5 mg/ml NHS reagent, then cross-linked with 1 mg antibody LD9E3. Please refer to Table 1 for abbreviations.

AE-labeled detection antibody: 1 mg Antibody LD7C1 was labelled with 0.25 mM AE in the 0.1 M Sodium hydrocarbonate solution, pH 9.0. The reaction of antibody and AE was performed in the dark for 12 h at room temperature. After desalting with a Sephadex G-25 column, the labeling efficiency was defined as the ratio of the molar concentration of AE to that of labeled LD7C1.

Commercial LAM antigen was used as the standard and serial diluted with PBS. This LAM reference standard was set as 0, 5,10,100,1000,10000 pg/ml. The measurement of LAM by the CLIA-LAM assay was processed as below: first, about 1.5 ml urine samples were mixed with the capture antibody magnetic bead for one hour. Magnetic beads were separated and added to a reaction cup of the Automated immunoassay analyzer SMART 500 S (Chongqing Keysmile Biological). From the second step, the assay was processed automatically inside the SMART500s analyzer. Briefly, the capturing antibody magnetic beads and antigen mixture was washed five times before reacting with the AE-labeled LD7C1 antibody for 15 min. In the third step, after 5 times washing, 100 ul pre-stimulation reagent, and 100 ul stimulation reagent were added to the cup immediately prior to detection. The light signal is measured by a photomultiplier as relative light unit (RLU). Based on the signal generated for the sample compared with those generated from the standard calibrators, an index value is determined [6].

### Clinical samples validation

After the establishment of the methodology and the determination of the cutoff value of CLIA-LAM assay. 87 patients with confirmed ATB patients (including 11 hematogenous disseminated TB patients), 19 LTBI patients and 104 healthy individual samples were tested for confirmation and analysis.

### Statistical analysis

SPSS Statistics 20.0 (IBM, Armonk, NY) was used for statistical analysis. The normality of data was analyzed with the use of the Kolmogorov-Smirnov test. Data with normal distributions is expressed as mean ± SD; data with skew distributions is expressed as median and interquartile ranges. Skew-distributed data was compared by means of the Mann-Whitney U test. The receiver operating characteristic (ROC) curve was performed to obtain a cutoff value. A value of *P* < 0.05 was considered statistically significant.

## Results

### Characterization of the LAM-specific rabbit mAbs

**Fig. 1:**
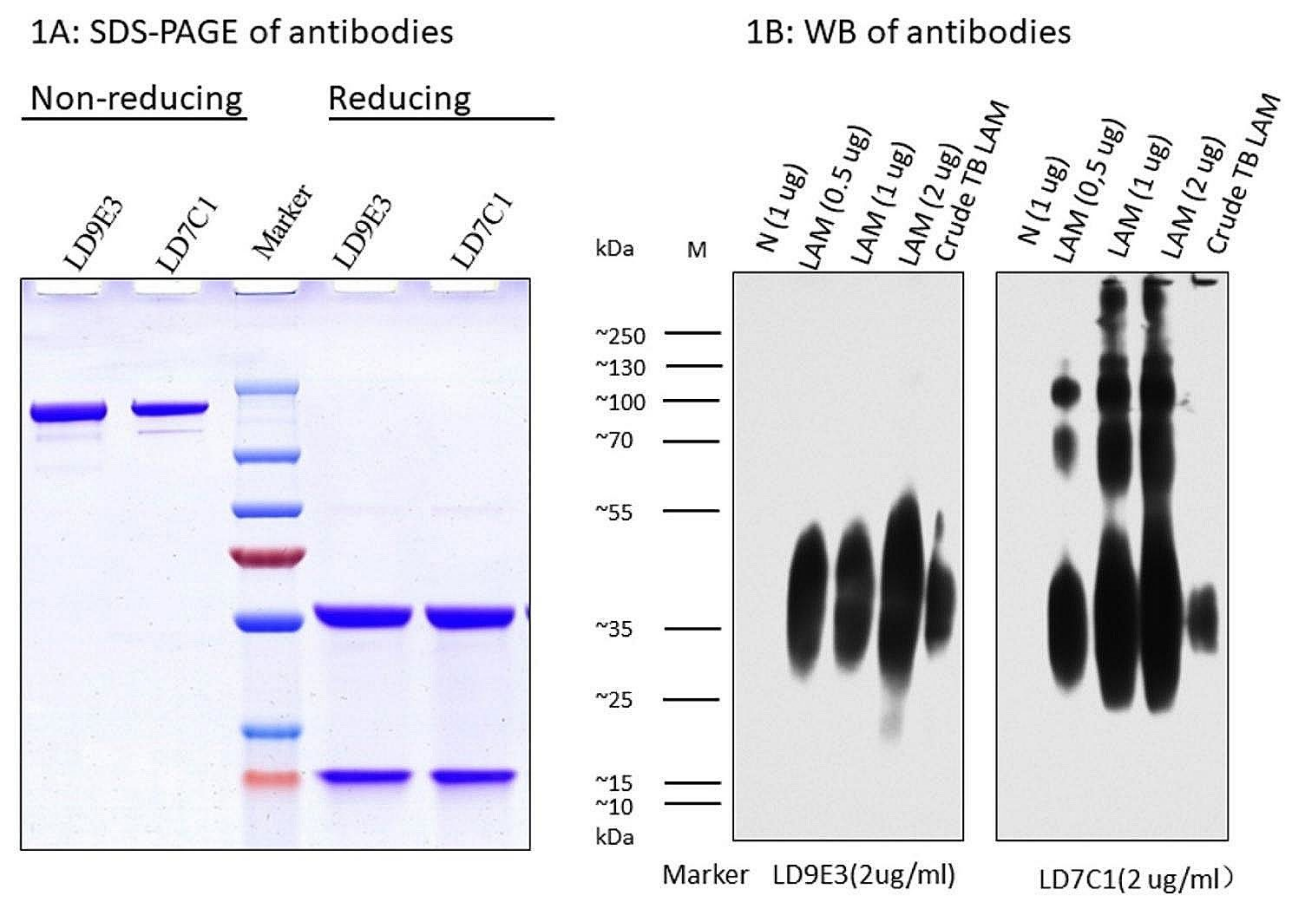
SDS-PAGE and western blot analysis of purified LAM-specific rabbit mAbs. (**A**) Recombinant antibodies LD9E3 and LD7C1 were tested by SDS–PAGE under non-reducing and reducing conditions. (**B**) Reactivities of rabbit anti-LAM mAbs to LAM antigens were tested by western blotting with 0.5, 1, 2 ug of LAM antigen and 10 ul crude TB LAM, respectively. M: Prestained protein ladder (10 to 250 kDa) was used. After electrophoretic transfer, a molecular weight marker was drawn on the membrane with the sole purpose of indicating the molecular size. N: negative control lane, bacterial E. coli lysate prepared in the same manner as the crude TB Lam sample

Rabbit antibodies were expressed and purified from 293T cells. The purity of the recombinant LD7C1 and LD9E3 antibodies were tested by SDS–PAGE. Rabbit anti-LAM mAbs under non-reducing conditions showed single band (150 kDa), and two bands (25 kDa and 50 kDa) under reducing conditions (Fig. 1a). Reactivities of rabbit anti-LAM mAbs to the LAM antigens were tested by western blotting with 0.5, 1 and 2 ug of LAM standards and 10 ul crude TB LAM, respectively. The reported molecular weight of LAM has been shown to vary, with some sources indicating a weight of approximately 17.4 kDa while others suggest a weight of around 35 kDa. The results of the Western blot analysis indicate that the binding specificity of LD9E3 is superior to that of LD7C1, as LD9E3 recognized only a single smear band in the 30–50 kDa range. LD7C1 recognized some larger-size molecules with unclear identity in the LAM antigen. Interestingly, both LD9E3 and LD7C1 only recognized one band in the crude TB extract (Fig. 1b).

### Antibodies affinity kinetic test

**Fig. 2:**
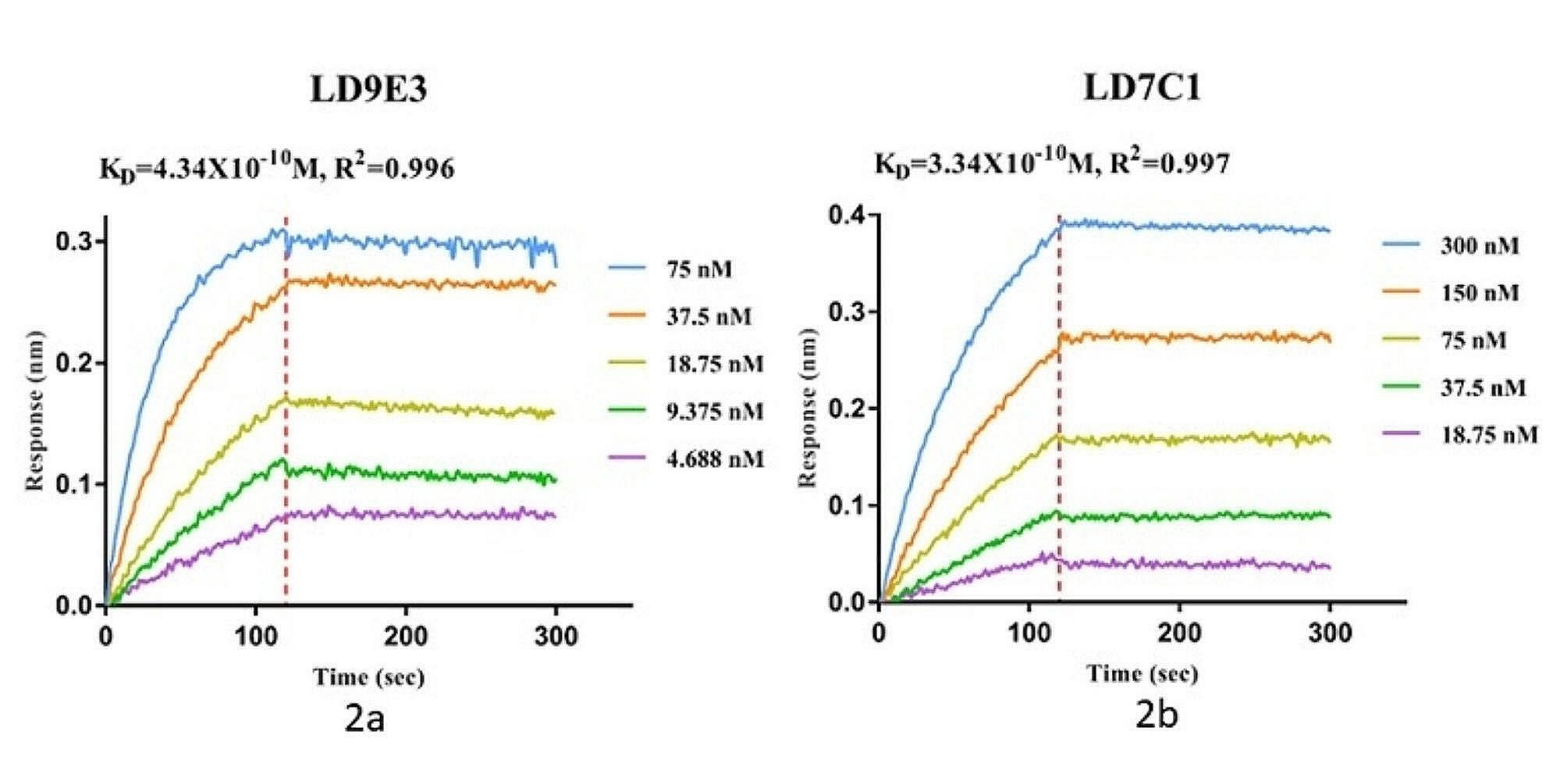
ForteBio Octet determination of the affinities of the (**A**) LD9E3 and (**B**) LD7C1 toward LAM. A sensorgram showing LAM on the aminopropylsilane sensors binding to different concentrations of rabbit mAbs. Saline buffer was used as the negative control. The binding affinity parameter KD was calculated. R2 is the coefficient of determination for estimating the goodness of the curve fit, as reported by ForteBio Data Analysis Software 9.0

The affinity of the mAbs for LAM was determined using ForteBio Octet system assays (Fig. 2). After the raw data was analyzed, the binding affinities of LD7C1 and LD9E3 for LAM were determined (R2 = 0.997 and R2 = 0.996, respectively), and the KD values were measured as 3.34 × 10–10 M and 4.34 × 10–10 M, respectively.

### Cutoff value determination of CLIA LAM assay

From 2021 to 2022, Foshan Fourth People’s Hospital enrolled a total of 146 patients with pulmonary tuberculosis, with 9 cases excluded due to tuberculosis treatment, resulting in a final inclusion of 137 samples. A total of 172 individuals without symptoms related to tuberculosis and no history of tuberculosis contact were recruited from the physical examination department as healthy controls. Three cases with insufficient samples were excluded, leaving a final inclusion of 169 individuals for the healthy control group.

The first 50 confirmed ATB samples, and the initial 65 samples from healthy individuals, were tested for the purpose of determining the cutoff.

Characteristics of the TB group and the healthy group samples are shown in Table 2. Test result was provided in Supplement Table 1.

**Table 2:** Characteristics of cutoff value determination samples

Characteristics	N	Age, years ($\overline{\varvec{X}}\pm \varvec{S}$)	Male, N (%)
Healthy	65	52.5 ± 21.7	46 (71%)
TB	50	56.3 ± 15.4	43 (86%)

A total of 50 patients with confirmed tuberculosis (TB) and 65 healthy individual samples were tested for cutoff value determination and assay development

**Fig. 3:**
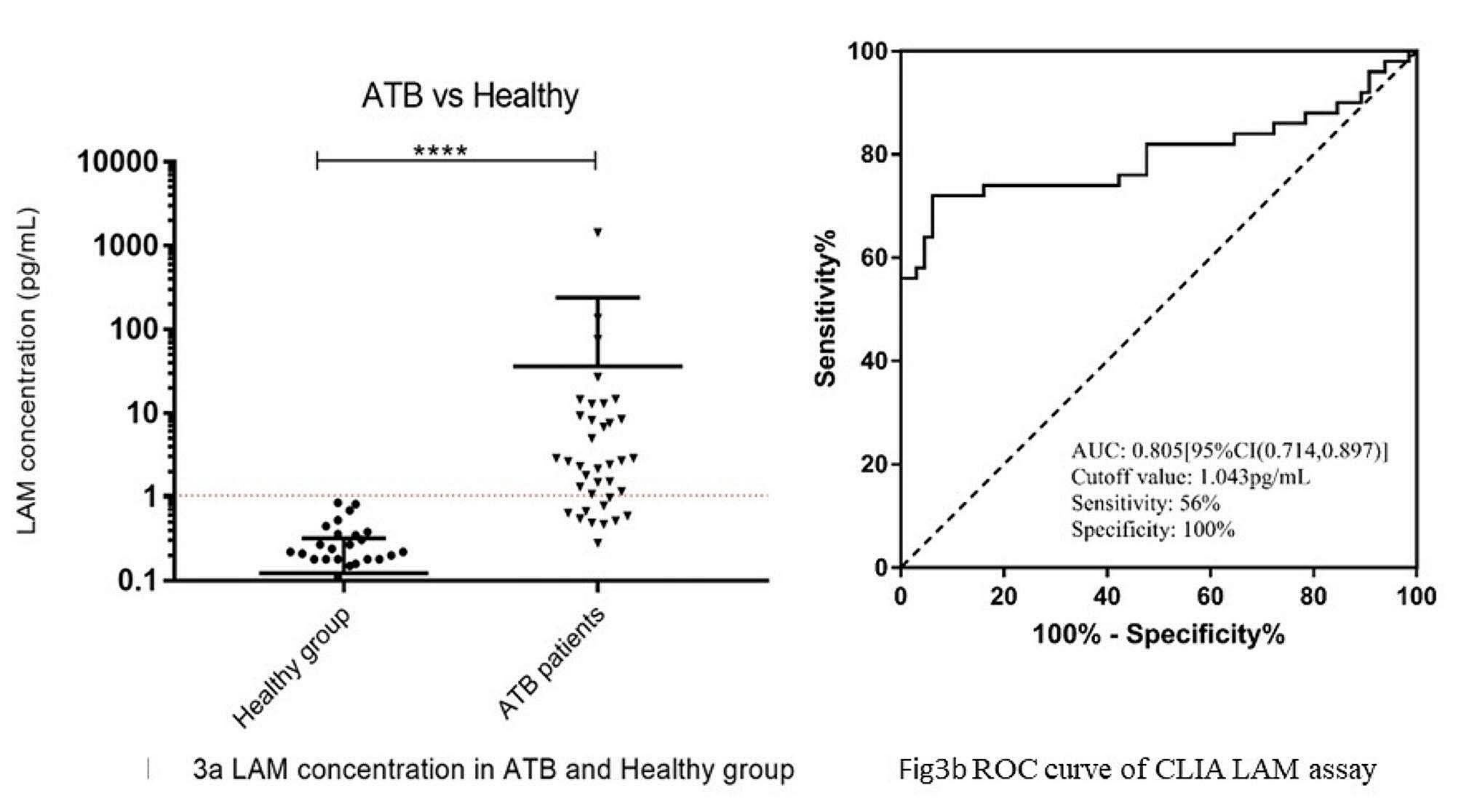
LAM concentration in ATB and healthy group, ROC curve of CLIA LAM assay. **3a**: LAM concentration in ATB and healthy group comparison: the average concentration of LAM in the ATB patient’s urine sample was 36.22 pg/mL. The average concentration of LAM in the healthy group urine sample was 0.12 pg/mL. A statistically significant difference in the LAM concentrations was observed between ATB and healthy groups (*p* < 0.0001). **3b**: ROC curve of CLIA LAM assay, when the cutoff value was set at 1.043 pg/mL, the sensitivity of CLIA-LAM was 56.0% and the specificity was 100%

The average concentration of LAM in the ATB patient’s urine sample was 36.22 pg/mL. The average concentration of LAM in the healthy group urine sample was 0.12 pg/mL. A statistically significant difference in the LAM concentrations was observed between ATB and healthy groups (*p* < 0.0001) (Fig. 3a).

The ROC curve was drawn according to the clinical diagnosis, and the AUC was 0.805 [95%CI (0.714, 0.897)]. When the cutoff value was 1.043 (pg/mL), the sensitivity of CLIA-LAM was 56.0% [95%CI (41.3%, 70.0%)] and the specificity was 100% [95%CI (94.5%, 100%)] (Fig. 3b).

### Assay validation

Of the 137 patients with active tuberculosis (ATB), the most recent 87 ATB cases, the latest 104 samples from healthy individuals, and 19 samples from individuals with latent tuberculosis infection (LTBI) underwent testing for confirmation, validation, and analysis. Among the ATB cases, 11 instances of hematogenous disseminated tuberculosis were clinically confirmed. Nineteen cases of latent tuberculosis infection (LTBI) were obtained from suspected TB patients, specifically outpatient individuals, who tested negative for tuberculosis but positive for Interferon-Gamma Release Assay (IGRA).

Characteristics of the TB group and the healthy group are shown in Table 3. Test result was provided in Supplement Table 2.

**Table 3:** Characteristics of verification samples

Characteristics	N	Age, years ($\overline{\varvec{X}}\pm \varvec{S}$)	Male, n (%)
Healthy	104	36.6 ± 12.9	48 (46%)
LTBI	19	41.7 ± 9.4	4 (21%)
Total active TB (ATB)	87	47.2 ± 23.2	56 (64%)
Hematogenous disseminated TB	11	51.1 ± 19.9	9 (82%)

A total of 87 patients with confirmed TB, 19 LTBI patients, and 104 healthy individuals were recruited for CLIA-LAM assay validation

**Fig. 4:**
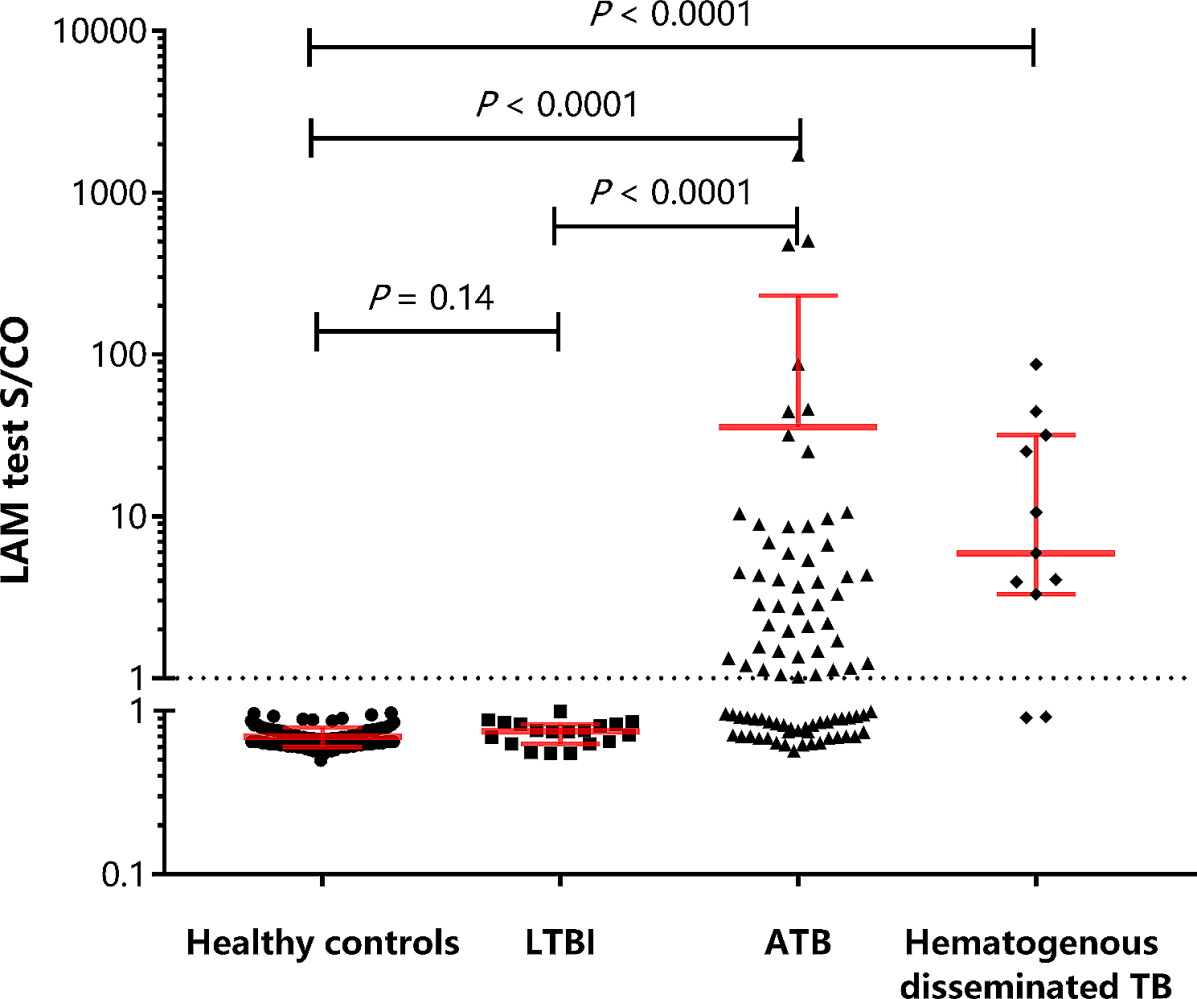
The comparison of LAM concentration of healthy people, LTBI, TB patients, and hematogenous disseminated TB patients. We compared the levels of LAM concentration in 87 TB patients (including 11 with hematogenous disseminated TB), 104 healthy individuals, and 19 latent TB infection (LTBI) samples. The average LAM concentration for TB patients was 35.66 (pg/mL), while the average for healthy controls was 0.70 (pg/mL). The average LAM concentration for hematogenous disseminated TB patients was 19.86 (pg/mL). The average LAM concentration for LTBI samples was 0.74 (pg/mL). We found a significant difference of LAM levels between TB patients and healthy controls (*P* < 0.0001), between hematogenous disseminated TB patients and healthy controls (*P* < 0.0001), and between LTBI and TB patients (*P* < 0.0001). However, there was no significant difference in LAM levels between LTBI and healthy controls (*P* = 0.14). The positive detection rate for hematogenous disseminated TB patients was 81.8% (9/11)

The average LAM concentration (pg/mL) of the TB patients was significantly higher than the healthy controls (*P* < 0.0001) (Fig. 4). The average LAM concentration for TB patients was 35.66 (pg/mL), while the average for healthy controls was 0.70 (pg/mL). The average LAM concentration for hematogenous disseminated TB patients was 19.86 (pg/mL). The average LAM concentration for LTBI samples was 0.74 (pg/mL). We found a significant difference of LAM levels between TB patients and healthy controls (*P* < 0.0001), between hematogenous disseminated TB patients and healthy controls (*P* < 0.0001), and between LTBI and TB patients (*P* < 0.0001). However, there was no significant difference in LAM levels between LTBI and healthy controls (*P* = 0.14). The positive detection rate for hematogenous disseminated TB patients was 81.8% (9/11).

### Diagnostic efficacy

Taking clinical diagnosis as the gold standard, the positive percent agreement (PPA), negative percent agreement (NPA), and total coincidence rate of LAM in the diagnosis of TB were 55.2% [95%CI(44.13%~65.85%)], 100.0% [95%CI(96.52%~100.00%)], and 72.7% [95%CI (64.65%~79.83%)], respectively (Table 4).

**Table 4:** The diagnostic performance of CLIA LAM assay

Methods	Results	CRS		Sensitivity		Specificity		PPV		NPV		Kappa		
		TB	Non-TB											
LAM	positive	48	0		55.2%		100%		100%		72.7%		0.573	
	negative	39	104											
					(44.13%~65.85%)		(96.52%~100.00%)		(92.60%~100.00)		(64.65%~79.83%)			

PPV, positive predictive value; NPV, negative predictive value. Sensitivity = True Positive Cases / (True Positive Cases + False Negative Cases) × 100%; Specificity = True Negative Cases / (True Negative Cases + False Positive Cases) × 100%; PPV = True Positive Cases / (True Positive Cases + False Positive Cases) × 100%; NPV = True Negative Cases / (True Negative Cases + False Negative Cases) × 100%;

## Discussion

In this study, we address sensitivity and specificity concerns through the synergistic combination of high-affinity antibodies, chemiluminescent immunoassay (CLIA), and magnetic bead concentration steps in the CLIA-LAM assay. This innovative approach has yielded significant progress, achieving a remarkable sensitivity of 55.2% [95%CI (44.13%~65.85%)] and an impressive specificity of 100% [95%CI (96.52%~100.00%)] in active TB patients.

Due to the advantage of LAM assays being the non-invasive nature of urine collection, easily obtainable from patients, the use of urinary lipoarabinomannan (LAM) assays for TB diagnosis has gained increasing attention and undergone significant advancements in recent years [7–15]. The AlereLAM assay has shown low sensitivity in HIV-negative TB patients, typically ranging from only 10 to 20%. To improve its diagnostic accuracy, various methods have been explored. One promising approach involves concentrating the sample, which has been shown to enhance sensitivity in several studies. For instance, Paris et al. reported a 95% sensitivity at 80% specificity by detecting urine LAM down to 14 pg/mL after concentration [16], while Shapiro et al. achieved a 52% sensitivity at 67% specificity in HIV-negative TB-positive individuals through urine sample concentration [17]. Connelly et al. used a concentration step before the lateral flow assay and reported a sensitivity of 60% at 80% specificity [18], and Hamasur et al. enriched LAM using magnetic particles to achieve a sensitivity of 65.5% at 84% specificity [19]. Although these approaches have improved assay sensitivity, they have often come at the cost of specificity, which has remained below 85%. Therefore, while sample concentration can enhance sensitivity, it alone is not sufficient to resolve the issue.

The Fujifilm SILVAMP TB LAM (FujiLAM; Fujifilm, Tokyo, Japan) point-of-care (POC) assay uses two high-affinity antibodies and a silver amplification step to improve sensitivity to 66.7% and specificity to 96.2% in combined HIV-infected and uninfected group [20, 21, 22]. However, the silver amplification step alone may result in non-specific outcomes and operational difficulties, and this study included only a relatively small number of samples.

The CLIA-LAM assay employs a one-hour pretreatment process to concentrate urine lam with magnetic beads coated with capture antibodies, followed by fully automated processing inside the SMART500s analyzer. This approach ensures high precision and reproducibility, while also reducing the risk of human error. The assay requires minimal hands-on time, taking only 5 min, and the total diagnostic time is approximately 1.5 h. With machine operation and result reporting taking just 18 min, the CLIA-LAM assay provides a rapid turnaround time.

This study attempted to address the sensitivity and specificity issues of the LAM assay from three different angles. Firstly, high-affinity rabbit monoclonal antibodies (LD7C1 and LD9E3) were selected based on their superior affinity compared to mouse antibodies [22]. These antibodies were screened using the LAM-immune rabbit and phage-display method. Secondly, chemiluminescent immunoassay (CLIA) was chosen due to its wide dynamic range, high signal intensity, low interfering emissions, and automation compared to traditional ELISA and point-of-care testing (POCT) methods. Thirdly, magnetic bead conjugated antibodies were used to capture LAM antigens from larger sample volumes. When these approaches were combined, the sensitivity and specificity of the CLIA-LAM assay was greatly improved in active TB patients. Unfortunately, a direct comparison between the AlereLAM assay and the Fujifilm SILVAMP TB LAM product was not feasible due to the unavailability of both products for evaluation.

It is worth noting that all of the TB patients in this study were HIV-negative. Previous research has demonstrated that HIV/TB co-infected patients with immunosuppression and disseminated TB tend to have higher LAM concentrations in their urine [23, 24]. The major question regarding the LAM assay is whether it can be used as a reliable TB biomarker. Specifically, it is unclear whether the LAM levels in the urine of HIV negative patients- are simply too low to be measured by the current test [8, 25, 26].. Our findings revealed that urine LAM concentrations in HIV-negative TB patients were generally low. Specifically, in 39 out of 47 positive cases, LAM concentrations ranged from 1 to 20 pg/mL, which could only be detected by our highly sensitive CLIA-LAM assay (Data from supplement Table 1).

The LAM antigen concentration cutoff for this CLIA-LAM assay was notably lower than the cutoff reported by Broger et al. of approximately 11 pg/mL. Broger et al. found that 93% of patients had detectable levels of LAM antigen, ranging from 12 pg/mL to 90,000 pg/mL [7, 8]. The difference in cutoffs may be attributed to variations in patient populations and LAM antigen purity and quantification methods. In the western blot result, the commercial LAM antigen showed a smear band between 30 and 50 kDa (Fig. 1b). We observed that the LD7C1 antibody recognized some larger molecules with unknown identity in the commercial LAM antigen that were not present in the crude TB extract. This suggests that LAM smear, multimers, or LAM-related complexes might have formed during the purification process. In the future, the development of a high-purity, accurately quantified, and traceable LAM antigen standard may be crucial for the next generation of LAM assays.

In the samples from healthy individuals, LAM antigens were less than 1 pg/mL even after magnetic antibody concentration step (Fig. 4). Given the nearly 100% specificity and the fact that samples from latent TB infection (LTBI) patients had zero positives, this CLIA-LAM assay may be useful for confirming active TB (ATB) diagnosis and for distinguishing between LTBI and ATB. However, it should be noted that the sample size (*n* = 19) was small and this conclusion requires further investigation.

Disseminated TB is a life-threatening condition if diagnosis and treatment are delayed. In the 11 hematogenous disseminated TB patients in this study, the CLIA-LAM assay demonstrated high sensitivity (81.8%), providing promising results for future research. It is yet to be determined whether this CLIA-LAM assay can also be used to diagnose extrapulmonary TB (EPTB), pediatric patients [27, 28], and ICU patients, and further investigation is required to address these questions.

## Conclusion

In summary, this study underscores the potential of the CLIA-LAM assay in Tuberculosis (TB) diagnosis through the integration of high-affinity antibodies, chemiluminescent immunoassay, and magnetic bead concentration. Nevertheless, additional research with larger sample sizes and investigation into its applicability across diverse patient groups are imperative for comprehensive validation.

### Electronic supplementary material

Below is the link to the electronic supplementary material.

Supplementary Material 1

Supplementary Material 2

Supplementary Material 3

Supplementary Material 4

Supplementary Material 5

## Data Availability

The authors confirm that the data supporting the findings of this study are available within the article and/or its supplementary materials.
