# Supplementary material for: Improving the diagnosis of active tuberculosis: a novel approach using magnetic particle-based chemiluminescence LAM assay
Source: BMC Pulm Med. 2024 Feb 27;24:100. doi: 10.1186/s12890-024-02893-2 (PMC10898140; doi:10.1186/s12890-024-02893-2)
Supplement: Supplementary file 1 — Supplementary Material 1 [file 12890_2024_2893_MOESM1_ESM.pdf]

Table S1: Sample Information for Calculating the Cutoff of LAM-CLIA Assay

This table presents the sample information for calculating the cutoff of the LAM-CLIA assay, including the number of patients, their sex, age, clinical diagnosis result, and LAM test results. Clinical diagnosis result refers to the patient's confirmed or suspected diagnosis, while LAM test results

NO.	Sex	Age	Clinical diagnosis	LAM test (RLUs)	LAM test(S/CO)	LAM test(pg/ml)	Gene Xpert MTB/RIF	sputum smear	sputum culture
H001	Male	33	Healthy	824	0.76	0.01	/	/	/
H002	Male	40	Healthy	858	0.79	0.15	/	/	/
H003	Male	89	Healthy	866	0.80	0.18	/	/	/
H004	Female	32	Healthy	877	0.81	0.22	/	/	/
H005	Female	56	Healthy	866	0.80	0.18	/	/	/
H006	Female	39	Healthy	873	0.81	0.20	/	/	/
H007	Female	56	Healthy	876	0.81	0.22	/	/	/
H008	Female	37	Healthy	866	0.80	0.18	/	/	/
H009	Male	71	Healthy	784	0.72	0.00	/	/	/
H010	Female	74	Healthy	695	0.64	0.00	/	/	/
H011	Male	71	Healthy	834	0.77	0.05	/	/	/
H012	Female	33	Healthy	839	0.77	0.07	/	/	/
H013	Female	23	Healthy	720	0.66	0.00	/	/	/
H014	Male	18	Healthy	840	0.78	0.07	/	/	/
H015	Male	56	Healthy	866	0.80	0.18	/	/	/
H016	Male	69	Healthy	796	0.73	0.00	/	/	/
H017	Male	87	Healthy	721	0.67	0.00	/	/	/
H018	Male	66	Healthy	786	0.73	0.00	/	/	/
H019	Male	25	Healthy	775	0.71	0.00	/	/	/
H020	Male	47	Healthy	719	0.66	0.00	/	/	/
H021	Female	52	Healthy	875	0.81	0.21	/	/	/
H022	Female	61	Healthy	669	0.62	0.00	/	/	/
H023	Male	46	Healthy	735	0.68	0.00	/	/	/
H024	Female	39	Healthy	793	0.73	0.00	/	/	/
H025	Female	88	Healthy	725	0.67	0.00	/	/	/
H026	Male	76	Healthy	685	0.63	0.00	/	/	/
H027	Male	25	Healthy	785	0.72	0.00	/	/	/
H028	Male	75	Healthy	751	0.69	0.00	/	/	/
H029	Male	80	Healthy	714	0.66	0.00	/	/	/
H030	Male	19	Healthy	660	0.61	0.00	/	/	/
H031	Male	25	Healthy	935	0.86	0.45	/	/	/
H032	Male	76	Healthy	911	0.84	0.36	/	/	/
H033	Male	75	Healthy	841	0.78	0.08	/	/	/
H034	Male	80	Healthy	849	0.78	0.11	/	/	/
H035	Male	67	Healthy	889	0.82	0.27	/	/	/
H036	Male	25	Healthy	867	0.80	0.18	/	/	/
H037	Female	24	Healthy	701	0.65	0.00	/	/	/
H038	Male	80	Healthy	996	0.92	0.69	/	/	/
H039	Male	54	Healthy	917	0.85	0.38	/	/	/
H040	Male	75	Healthy	1035	0.95	0.85	/	/	/
H041	Male	70	Healthy	900	0.83	0.31	/	/	/

H042	Male	43	Healthy	767	0.71	0.00	/	/	/
H043	Male	54	Healthy	890	0.82	0.27	/	/	/
H044	Male	54	Healthy	692	0.64	0.00	/	/	/
H045	Female	70	Healthy	652	0.60	0.00	/	/	/
H046	Male	88	Healthy	747	0.69	0.00	/	/	/
H047	Male	67	Healthy	1028	0.95	0.82	/	/	/
H048	Female	70	Healthy	818	0.75	0.00	/	/	/
H049	Female	61	Healthy	663	0.61	0.00	/	/	/
H050	Male	70	Healthy	862	0.79	0.16	/	/	/
H051	Male	46	Healthy	621	0.57	0.00	/	/	/
H052	Male	54	Healthy	686	0.63	0.00	/	/	/
H053	Male	88	Healthy	738	0.68	0.00	/	/	/
H054	Male	43	Healthy	768	0.71	0.00	/	/	/
H055	Female	39	Healthy	798	0.74	0.00	/	/	/
H056	Male	53	Healthy	700	0.65	0.00	/	/	/
H057	Female	19	Healthy	654	0.60	0.00	/	/	/
H058	Male	57	Healthy	828	0.76	0.02	/	/	/
H059	Male	49	Healthy	733	0.68	0.00	/	/	/
H060	Female	38	Healthy	955	0.88	0.53	/	/	/
H061	Male	10	Healthy	881	0.81	0.24	/	/	/
H062	Male	28	Healthy	760	0.70	0.00	/	/	/
H063	Male	33	Healthy	679	0.63	0.00	/	/	/
H064	Male	14	Healthy	910	0.84	0.35	/	/	/
H065	Male	32	Healthy	771	0.71	0.00	/	/	/
P001	Male	79	TB	4073	3.76	12.92	Negative	/	Negative
P002	Female	78	TB	893	0.82	0.28	Postive	Postive	Postive
P003	Male	59	TB	2955	2.73	8.48	Negative	Postive	/
P004	Male	70	TB	715	0.66	0.00	Postive	Negative	/
P005	Male	50	TB	724	0.67	0.00	/	Negative	/
P006	Male	34	TB	990	0.91	0.67	Negative	Negative	Postive
P007	Male	28	TB	945	0.87	0.49	Postive	Negative	/
P008	Male	61	TB	1154	1.06	1.32	Negative	Postive	Postive
P009	Female	83	TB	1551	1.43	2.90	/	Negative	/
P010	Male	68	TB	2079	1.92	4.99	/	Negative	/
P011	Male	56	TB	20033	18.48	76.33	Negative	/	Negative
P012	Male	74	TB	656	0.61	0.00	Postive	/	Negative
P013	Male	55	TB	812	0.75	0.00	Negative	Negative	/
P014	Male	56	TB	35244	32.51	136.76	Negative	/	/
P015	Male	56	TB	2549	2.35	6.86	Postive	Negative	Negative
P016	Male	42	TB	362743	334.63	1437.91	/	Negative	/
P017	Male	53	TB	672	0.62	0.00	Negative	Negative	/
P018	Male	53	TB	940	0.87	0.47	Postive	Negative	Negative
P019	Male	58	TB	1090	1.01	1.07	Negative	Postive	/
P020	Male	73	TB	1113	1.03	1.16	Postive	Negative	/
P021	Male	72	TB	631	0.58	0.00	Postive	Postive	/
P022	Male	20	TB	1486	1.37	2.64	Postive	Negative	/
P023	Male	32	TB	4510	4.16	14.65	/	/	Negative
P024	Male	71	TB	801	0.74	0.00	Negative	/	/
P025	Male	59	TB	689	0.64	0.00	Postive	Negative	Postive
P026	Female	49	TB	1274	1.18	1.80	Postive	/	/
P027	Female	57	TB	1546	1.43	2.88	Negative	/	/
P028	Male	49	TB	1198	1.10	1.49	Postive	Negative	Negative
P029	Male	54	TB	687	0.63	0.00	Postive	Postive	/

P030	Male	61	TB	2743	2.53	7.63	/	Negative	/
P031	Male	61	TB	952	0.88	0.52	Postive	Postive	/
P032	Male	66	TB	1018	0.94	0.78	/	Negative	/
P033	Male	46	TB	3174	2.93	9.34	Negative	Negative	/
P034	Male	65	TB	1503	1.39	2.71	Negative	/	/
P035	Male	80	TB	959	0.88	0.55	Postive	Postive	/
P036	Female	60	TB	801	0.74	0.00	/	Postive	/
P037	Male	71	TB	7620	7.03	27.01	Postive	Postive	Negative
P038	Male	47	TB	1431	1.32	2.42	Postive	/	/
P039	Male	68	TB	1407	1.30	2.32	Postive	Negative	/
P040	Female	62	TB	4524	4.17	14.71	/	/	Negative
P041	Male	43	TB	677	0.62	0.00	/	/	Postive
P042	Male	68	TB	1067	0.98	0.98	Postive	Negative	/
P043	Male	16	TB	971	0.90	0.59	Postive	/	/
P044	Male	53	TB	753	0.70	0.00	Postive	/	Negative
P045	Male	47	TB	830	0.77	0.03	/	Negative	/
P046	Male	29	TB	982	0.91	0.64	Negative	Negative	/
P047	Female	56	TB	1205	1.11	1.52	Postive	Postive	/
P048	Male	33	TB	1365	1.26	2.16	Postive	Postive	Postive
P049	Male	64	TB	2887	2.66	8.21	/	/	Negative
P050	Male	71	TB	4083	3.77	12.96	Postive	Negative	Negative

LAM (pg/ml)	LAM test (RLUs)
0	698
1	1073
10	3476
100	25978
