# Supplementary material for: Improving the diagnosis of active tuberculosis: a novel approach using magnetic particle-based chemiluminescence LAM assay
Source: BMC Pulm Med. 2024 Feb 27;24:100. doi: 10.1186/s12890-024-02893-2 (PMC10898140; doi:10.1186/s12890-024-02893-2)
Supplement: Supplementary file 2 — Supplementary Material 2 [file 12890_2024_2893_MOESM2_ESM.pdf]

Table S2: Sample Information for Confirming the Performance of LAM
This table provides sample information for confirming the performance of the LAM-CLIA assay, including the number of patients, their sex, age, and LAM test results. The LAM test results indicate the LAM-CLIA assay results for each patient sample, with positive or negative results confirmed by other diagnostic methods.

sampl	Sex	Age	Clinical diagnosis	LAM test (RLUs)	LAM test(S/CO)	LAM test(pg/ml)	Gene Xpert MTB/RIF	sputum smear	sputum culture
H066	Female	38	Healthy	865	0.80	0.17	/	/	/
H067	Female	50	Healthy	700	0.65	0.00	/	/	/
H068	Male	44	Healthy	842	0.78	0.08	/	/	/
H069	Male	25	Healthy	546	0.50	0.00	/	/	/
H070	Male	45	Healthy	758	0.70	0.00	/	/	/
H071	Female	34	Healthy	777	0.72	0.00	/	/	/
H072	Male	36	Healthy	886	0.82	0.26	/	/	/
H073	Male	63	Healthy	665	0.61	0.00	/	/	/
H074	Male	28	Healthy	706	0.65	0.00	/	/	/
H075	Male	38	Healthy	818	0.76	0.00	/	/	/
H076	Male	64	Healthy	709	0.65	0.00	/	/	/
H077	Male	34	Healthy	814	0.75	0.00	/	/	/
H078	Male	44	Healthy	600	0.55	0.00	/	/	/
H079	Male	27	Healthy	633	0.58	0.00	/	/	/
H080	Female	52	Healthy	620	0.57	0.00	/	/	/
H081	Female	38	Healthy	860	0.79	0.15	/	/	/
H082	Female	60	Healthy	700	0.65	0.00	/	/	/
H083	Male	33	Healthy	658	0.61	0.00	/	/	/
H084	Male	28	Healthy	724	0.67	0.00	/	/	/
H085	Male	27	Healthy	614	0.57	0.00	/	/	/
H086	Male	47	Healthy	680	0.63	0.00	/	/	/
H087	Male	58	Healthy	663	0.61	0.00	/	/	/
H088	Male	41	Healthy	727	0.67	0.00	/	/	/
H089	Female	60	Healthy	678	0.63	0.00	/	/	/
H090	Female	46	Healthy	697	0.64	0.00	/	/	/
H091	Female	55	Healthy	745	0.69	0.00	/	/	/
H092	Female	36	Healthy	674	0.62	0.00	/	/	/
H093	Female	58	Healthy	689	0.64	0.00	/	/	/
H094	Female	10	Healthy	647	0.60	0.00	/	/	/
H095	Female	33	Healthy	713	0.66	0.00	/	/	/
H096	Female	26	Healthy	745	0.69	0.00	/	/	/
H097	Female	32	Healthy	682	0.63	0.00	/	/	/
H098	Female	25	Healthy	743	0.68	0.00	/	/	/
H099	Female	52	Healthy	864	0.80	0.17	/	/	/
H100	Female	37	Healthy	740	0.68	0.00	/	/	/
H101	Male	27	Healthy	944	0.87	0.49	/	/	/
H102	Male	25	Healthy	680	0.63	0.00	/	/	/

H103	Male	32	Healthy	629	0.58	0.00	/	/	/
H104	Male	42	Healthy	709	0.65	0.00	/	/	/
H105	Male	31	Healthy	702	0.65	0.00	/	/	/
H106	Male	30	Healthy	743	0.69	0.00	/	/	/
H107	Male	28	Healthy	767	0.71	0.00	/	/	/
H108	Male	26	Healthy	785	0.72	0.00	/	/	/
H109	Male	32	Healthy	801	0.74	0.00	/	/	/
H110	Female	34	Healthy	615	0.57	0.00	/	/	/
H111	Female	41	Healthy	686	0.63	0.00	/	/	/
H112	Female	65	Healthy	718	0.66	0.00	/	/	/
H113	Female	37	Healthy	658	0.61	0.00	/	/	/
H114	Female	36	Healthy	961	0.89	0.55	/	/	/
H115	Female	38	Healthy	691	0.64	0.00	/	/	/
H116	Female	16	Healthy	716	0.66	0.00	/	/	/
H117	Female	40	Healthy	722	0.67	0.00	/	/	/
H118	Male	34	Healthy	713	0.66	0.00	/	/	/
H119	Female	37	Healthy	886	0.82	0.26	/	/	/
H120	Female	34	Healthy	684	0.63	0.00	/	/	/
H121	Female	62	Healthy	735	0.68	0.00	/	/	/
H122	Male	34	Healthy	768	0.71	0.00	/	/	/
H123	Male	29	Healthy	733	0.68	0.00	/	/	/
H124	Male	37	Healthy	644	0.59	0.00	/	/	/
H125	Male	32	Healthy	728	0.67	0.00	/	/	/
H126	Male	15	Healthy	742	0.68	0.00	/	/	/
H127	Male	32	Healthy	758	0.70	0.00	/	/	/
H128	Male	29	Healthy	979	0.90	0.63	/	/	/
H129	Male	36	Healthy	723	0.67	0.00	/	/	/
H130	Female	26	Healthy	767	0.71	0.00	/	/	/
H131	Female	28	Healthy	834	0.77	0.05	/	/	/
H132	Female	32	Healthy	810	0.75	0.00	/	/	/
H133	Female	25	Healthy	696	0.64	0.00	/	/	/
H134	Male	28	Healthy	1041	0.96	0.87	/	/	/
H135	Male	35	Healthy	647	0.60	0.00	/	/	/
H136	Male	33	Healthy	827	0.76	0.02	/	/	/
H137	Male	27	Healthy	947	0.87	0.50	/	/	/
H138	Male	27	Healthy	920	0.85	0.39	/	/	/
H139	Male	64	Healthy	768	0.71	0.00	/	/	/
H140	Female	26	Healthy	672	0.62	0.00	/	/	/
H141	Female	61	Healthy	853	0.79	0.12	/	/	/
H142	Female	25	Healthy	752	0.69	0.00	/	/	/
H143	Female	42	Healthy	835	0.77	0.05	/	/	/
H144	Male	27	Healthy	650	0.60	0.00	/	/	/
H145	Female	27	Healthy	658	0.61	0.00	/	/	/
H146	Female	67	Healthy	956	0.88	0.53	/	/	/
H147	Male	48	Healthy	670	0.62	0.00	/	/	/
H148	Female	39	Healthy	649	0.60	0.00	/	/	/
H149	Male	68	Healthy	745	0.69	0.00	/	/	/
H150	Female	35	Healthy	734	0.68	0.00	/	/	/
H151	Female	64	Healthy	705	0.65	0.00	/	/	/

H152	Female	38	Healthy	820	0.76	0.00	/	/	/
H153	Female	26	Healthy	829	0.77	0.03	/	/	/
H154	Female	20	Healthy	823	0.76	0.00	/	/	/
H155	Female	24	Healthy	837	0.77	0.06	/	/	/
H156	Male	24	Healthy	653	0.60	0.00	/	/	/
H157	Female	25	Healthy	722	0.67	0.00	/	/	/
H158	Female	41	Healthy	623	0.57	0.00	/	/	/
H159	Female	22	Healthy	707	0.65	0.00	/	/	/
H160	Female	24	Healthy	694	0.64	0.00	/	/	/
H161	Male	59	Healthy	812	0.75	0.00	/	/	/
H162	Male	38	Healthy	1027	0.95	0.81	/	/	/
H163	Female	25	Healthy	676	0.62	0.00	/	/	/
H164	Female	29	Healthy	885	0.82	0.25	/	/	/
H165	Female	24	Healthy	1050	0.97	0.91	/	/	/
H166	Female	36	Healthy	852	0.79	0.12	/	/	/
H167	Female	29	Healthy	842	0.78	0.08	/	/	/
H168	Female	22	Healthy	792	0.73	0.00	/	/	/
H169	Male	28	Healthy	1012	0.93	0.76	/	/	/
L001	Male	51	LTBI	601	0.55	0.00	/	/	/
L002	Female	41	LTBI	901	0.83	0.32	/	/	/
L003	Male	40	LTBI	602	0.55	0.00	/	/	/
L004	Female	52	LTBI	904	0.83	0.33	/	/	/
L005	Female	33	LTBI	877	0.81	0.22	/	/	/
L006	Female	42	LTBI	1073	0.99	1.00	/	/	/
L007	Female	54	LTBI	828	0.76	0.02	/	/	/
L008	Male	51	LTBI	745	0.69	0.00	/	/	/
L009	Male	55	LTBI	817	0.75	0.00	/	/	/
L010	Female	55	LTBI	822	0.76	0.00	/	/	/
L011	Female	43	LTBI	609	0.56	0.00	/	/	/
L012	Female	42	LTBI	765	0.71	0.00	/	/	/
L013	Female	28	LTBI	704	0.65	0.00	/	/	/
L014	Female	38	LTBI	683	0.63	0.00	/	/	/
L015	Female	27	LTBI	687	0.63	0.00	/	/	/
L016	Female	36	LTBI	937	0.86	0.46	/	/	/
L017	Female	41	LTBI	922	0.85	0.40	/	/	/
L018	Female	39	LTBI	953	0.88	0.52	/	/	/
L019	Female	25	LTBI	810	0.75	0.00	/	/	/
P051	Male	6	TB	1074	0.99	1.00	Postive	Negative	Negative
P052	Male	10	TB	4692	4.33	15.38	Negative	Negative	/
P053	Female	12	TB	1846796	1703.69	7334.02	Postive	Postive	/
P054	Male	12	TB	1139	1.05	1.26	Negative	Negative	/
P055	Female	13	TB	811	0.75	0.00	Negative	Postive	Negative
P056	Female	14	TB	690	0.64	0.00	Postive	Negative	Negative
P057	Male	14	TB	3997	3.69	12.62	Postive	Negative	/
P058	Female	14	TB	4704	4.34	15.42	/	Negative	/
P059	Male	14	TB	7206	6.65	25.37	Postive	Negative	/
P060	Male	14	TB	859	0.79	0.15	Postive	Negative	Negative
P061	Male	14	TB	959	0.89	0.55	Negative	Negative	/
P062	Male	15	TB	1220	1.13	1.58	Negative	/	/

P063	Male	18	TB	674	0.62	0.00	/	/	/
P064	Male	19	TB	986	0.91	0.65	Negative	Postive	Negative
P065	Female	23	TB	623	0.57	0.00	Negative	/	Negative
P066	Male	23	TB	2273	2.10	5.77	Postive	Postive	Postive
P067	Male	24	TB	675	0.62	0.00	Negative	Postive	/
P068	Female	24	TB	2121	1.96	5.16	Postive	Negative	/
P069	Male	25	TB	3020	2.79	8.74	/	/	/
P070	Male	26	TB	921	0.85	0.39	Negative	Negative	Postive
P071	Female	28	TB	762	0.70	0.00	Postive	Negative	/
P072	Male	28	TB	929	0.86	0.42	Negative	Postive	Postive
P073	Female	30	TB	744	0.69	0.00	/	Negative	/
P074	Female	30	TB	9369	8.64	33.96	/	Negative	/
P075	Female	32	TB	929	0.86	0.43	Negative	/	Negative
P076	Female	33	TB	887	0.82	0.26	Postive	/	Negative
P077	Male	33	TB	10505	9.69	38.47	Negative	Negative	/
P078	Female	37	TB	1030	0.95	0.83	Negative	/	/
P079	Female	39	TB	741	0.68	0.00	Postive	/	Negative
P080	Female	39	TB	1604	1.48	3.11	/	Negative	/
P081	Male	40	TB	886	0.82	0.25	Negative	Negative	/
P082	Male	43	TB	547559	505.13	2172.18	Postive	Postive	Negative
P083	Male	44	TB	806	0.74	0.00	Negative	Postive	/
P084	Male	46	TB	1446	1.33	2.48	Postive	Postive	/
P085	Female	46	TB	1600	1.48	3.09	Postive	Postive	/
P086	Male	49	TB	681	0.63	0.00	Postive	Postive	/
P087	Female	49	TB	1690	1.56	3.45	/	/	Negative
P088	Male	49	TB	5830	5.38	19.90	Negative	/	/
P089	Female	53	TB	739	0.68	0.00	Postive	Negative	Postive
P090	Male	53	TB	1302	1.20	1.91	Postive	/	/
P091	Male	54	TB	968	0.89	0.58	Negative	/	/
P092	Male	54	TB	1220	1.13	1.58	Postive	Postive	Negative
P093	Male	54	TB	2371	2.19	6.15	Postive	Postive	/
P094	Female	56	TB	814	0.75	0.00	/	Negative	/
P095	Male	56	TB	1473	1.36	2.59	Postive	Postive	/
P096	Female	58	TB	757	0.70	0.00	/	Negative	/
P097	Female	58	TB	851	0.78	0.11	Negative	Negative	/
P098	Male	59	TB	1849	1.71	4.08	Negative	/	/
P099	Female	61	TB	7463	6.88	26.39	Postive	Postive	/
P100	Male	62	TB	963	0.89	0.56	/	Postive	/
P101	Male	63	TB	1103	1.02	1.12	Postive	Negative	Negative
P102	Female	21	TB	1342	1.24	2.07	Postive	/	/
P103	Female	64	TB	771	0.71	0.00	Postive	Negative	/
P104	Female	66	TB	1140	1.05	1.26	/	/	/
P105	Male	67	TB	974	0.90	0.61	/	/	Postive
P106	Male	69	TB	11284	10.41	41.57	Postive	Negative	/
P107	Male	70	TB	912	0.84	0.36	Postive	/	/
P108	Female	70	TB	1034	0.95	0.84	Postive	/	/
P109	Male	70	TB	2933	2.71	8.39	/	/	/
P110	Male	70	TB	699	0.64	0.00	Negative	Negative	/
P111	Male	71	TB	764	0.70	0.00	Postive	Postive	/

P112	Male	71	TB	1007	0.93	0.74	Postive	Postive	Postive
P113	Male	71	TB	4580	4.23	14.93	/	/	/
P114	Female	74	TB	1039	0.96	0.86	Postive	Negative	/
P115	Female	74	TB	753	0.70	0.00	/	Postive	/
P116	Male	75	TB	2309	2.13	5.91	Postive	/	/
P117	Male	75	TB	516895	476.84	2050.35	Postive	/	/
P118	Male	76	TB	9435	8.70	34.22	Postive	Postive	/
P119	Male	77	TB	738	0.68	0.00	/	/	Postive
P120	Female	39	TB	4864	4.49	16.06	Negative	/	Postive
P121	Male	79	TB	1244	1.15	1.68	Negative	/	Postive
P122	Male	80	TB	49884	46.02	194.93	Negative	Negative	/
P123	Male	82	TB	9665	8.92	35.13	Negative	Postive	/
P124	Male	88	TB	3094	2.85	9.03	/	/	Negative
P125	Female	88	TB	3103	2.86	9.06	Postive	/	Negative
P126	Male	89	TB	828	0.76	0.03	Postive	Negative	/
P127	Male	12	Hematogenous	4266	3.94	13.68	Postive	/	Negative
P128	Male	53	Hematogenous	11460	10.57	42.27	/	Postive	/
P129	Male	53	Hematogenous	27268	25.16	105.07	Postive	/	Negative
P130	Male	67	Hematogenous	6414	5.92	22.22	Postive	/	Negative
P131	Male	52	Hematogenous	3586	3.31	10.98	Postive	/	/
P132	Male	63	Hematogenous	4402	4.06	14.23	Postive	Postive	Postive
P133	Male	78	Hematogenous	34512	31.84	133.85	Postive	Postive	Postive
P134	Female	63	Hematogenous	48254	44.52	188.45	Postive	Postive	Postive
P135	Male	58	Hematogenous	986	0.91	0.65	Postive	/	/
P136	Female	18	Hematogenous	998	0.92	0.70	Postive	/	Negative
P137	Male	45	Hematogenous	94653	87.32	372.79	/	/	Postive

CLIA LAM assay	Clinical diagnosis		Total
	Positive (+)	Negative (-)	
Positive (+)	48	0	48
Negative (-)	39	104	143
Total	87	104	191
