# Supplementary figures and images for: Improving the diagnosis of active tuberculosis: a novel approach using magnetic particle-based chemiluminescence LAM assay
Source: BMC Pulm Med. 2024 Feb 27;24:100. doi: 10.1186/s12890-024-02893-2 (PMC10898140; doi:10.1186/s12890-024-02893-2)

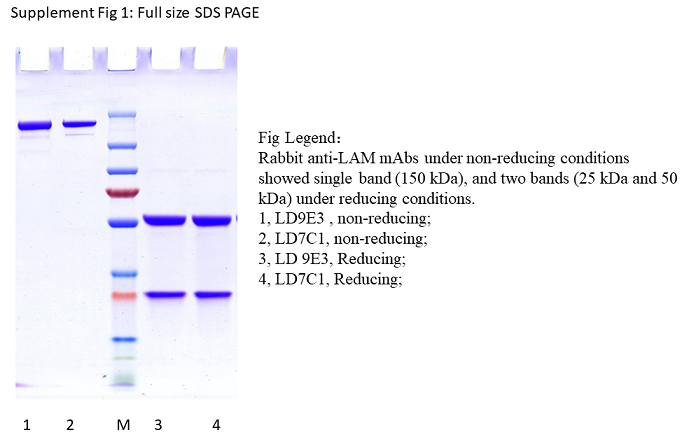

Supplement: Supplementary file 3 — Supplementary Material 3 [file 12890_2024_2893_MOESM3_ESM.tif]

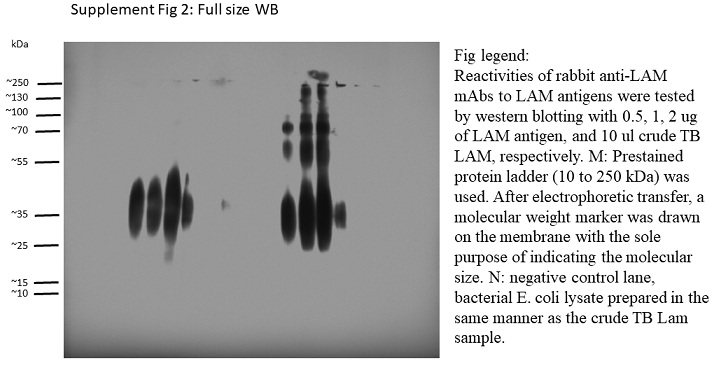

Supplement: Supplementary file 4 — Supplementary Material 4 [file 12890_2024_2893_MOESM4_ESM.tif]

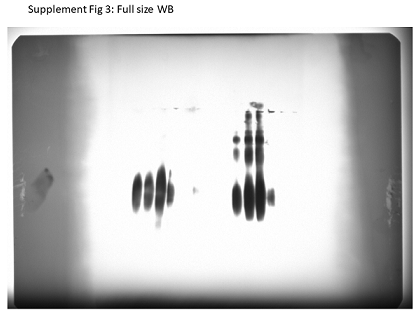

Supplement: Supplementary file 5 — Supplementary Material 5 [file 12890_2024_2893_MOESM5_ESM.tif]
